# Associations between medication beliefs and medication adherence among inpatients with chronic obstructive pulmonary disease: a cross-sectional survey

**DOI:** 10.3389/fpubh.2026.1934660

**Published:** 2026-09-16

**Authors:** Haiyan Li, Nannan Wang, Lu Zhang, Junfei Shi, Xiaoni Jia

**Affiliations:** 1 Department of Pharmacy, Xi’an People’s Hospital (Xi’an Fourth Hospital), Affiliated People’s Hospital of Northwest University, Xi’an, China; 2 Department of Pharmacy, The Second Affiliated Hospital of Inner Mongolia Medical University, Hohhot, China; 3 Department of Pharmacy, The First Affiliated Hospital of Xi’an Jiaotong University, Xi’an, China; 4 Department of Science and Education, Xi’an Mental Health Center, Xi’an, China

**Keywords:** chronic obstructive pulmonary disease (COPD), inhaled medication, inpatients, medication adherence, medication beliefs

## Abstract

**Background:**

Improving adherence to inhaled medication is crucial for optimal treatment outcomes and effective disease management in chronic obstructive pulmonary disease (COPD) patients. This study aimed to investigate adherence to inhaled medication and its association with medication beliefs among COPD patients in Xi’an, China.

**Methods:**

A cross-sectional study was conducted with 448 COPD inpatients recruited from two large tertiary hospitals in Xi’an, China, between May 1, 2024, and January 31, 2026. The Beliefs about Medicines Questionnaire Specific (BMQ-Specific) was used to assess inpatients’ beliefs about inhalers, and the Medication Adherence Report Scale (MARS-5) was employed to evaluate adherence to inhaled medication. Logistic regression analyses were performed to examine the associations between independent variables and medication non-adherence. Three sensitivity analyses were conducted in this study to confirm the model’s reliability. Model discrimination was assessed using ROC analysis, and internal validation was performed using 1,000 bootstrap resamples.

**Results:**

A total of 266 (59.4%) inpatients were non-adherent to inhaled medication. Overall, 243 (54.2%) inpatients expressed strong necessity beliefs about inhaled medication, while 203 (45.3%) held strong concern beliefs. Multivariable logistic regression analysis revealed that inpatient who expressed weaker necessity beliefs about inhaled medication had 4.79-fold odds (95% CI = 3.044–7.520; *p* < 0.001) of non-adherence compared with those who expressed stronger necessity beliefs. Inpatient who received dual therapy with LABA-ICS had 2.00-fold odds (95% CI = 1.027–3.900; *p* = 0.042) of non-adherence compared with those who received triple therapy with LAMA-LABA-ICS, whereas inpatient who was unemployed or retired was more likely to be adherent to inhaled medication (adjusted OR 0.534 [0.320, 0.889]; *p* = 0.016) than those in physical labor occupations. The association between necessity beliefs and medication adherence remained statistically significant upon validation in three sensitivity analyses.

**Conclusion:**

The relatively low adherence to inhaled medication among inpatients with COPD in Northwestern China highlights the urgent need for effective strategies to improve adherence. These findings suggest a potential clinical implication wherein pharmacists and nurses could play a supportive role in promoting medication adherence among patients with COPD.

## 1 Introduction

Chronic obstructive pulmonary disease (COPD), which is a prevalent and progressive lung disease characterized by persistent respiratory symptoms and airflow limitation, is the third leading cause of death worldwide (1). In China, the overall prevalence of spirometry-defined COPD was 8.6%, affecting approximately 100 million people aged 20 years or older in 2015 (2). As the country with the highest number of COPD cases worldwide (3), China accounted for approximately 25% of the global COPD population, imposing a considerable economic burden on the country (4). The burden of COPD is expected to increase dramatically in the coming decades due to the rapid aging of the Chinese population (4). Thus, increased attention and targeted interventions are warranted to address the significant health and economic challenges posed by this disease (5).

Inhalation therapy, as the cornerstone of COPD treatment and management (6), has been proved particularly effective in symptom management during the stable phase of the disease (7, 8). Adherence to medication can be defined as the degree to which use of medication by the patient corresponds with the prescribed regimen (9). Patients who regularly and consistently follow the prescribed regimen demonstrate adherent use. The most obvious form of nonadherence is chronic under-use, i.e., the patient consistently uses less medication than is prescribed (9). Medication adherence to inhaler therapy is important for controlling disease progression and treatment costs in patients with COPD (7, 10). However, medication adherence among individuals with COPD is notably suboptimal (7, 11–13), ranging between 20 and 50% (14). Non-adherence to inhaler therapy exacerbates symptoms and increases the frequency of hospital admissions (12).

Previous research has shown that medication beliefs are significantly associated with adherence to inhaled medications (7, 11–13, 15). A better understanding of the associations between medication beliefs and medication adherence could be crucial for improving disease outcomes (11, 16–18). Several studies have discussed the relationships between medication beliefs and medication adherence among patients with asthma (19–21). However, there is limited literature examining medication adherence among COPD patients in Northwestern China. Additionally, the associations between medication beliefs and adherence to inhaled medications among patients with COPD remain insufficiently investigated. As the largest city in Northwest China and the capital of Shaanxi Province, Xi’an differs significantly from other Chinese regions in racial composition, cultural background, and healthcare resources. All these factors may contribute to the associations between medication beliefs and medication adherence among individuals with COPD. This study aimed to investigate adherence to inhaled medication and its association with medication beliefs among COPD patients in Xi’an, China.

## 2 Methods

### 2.1 Study design and setting

A cross-sectional, questionnaire-based survey was conducted with 448 adult COPD inpatients admitted to the respiratory wards of two large tertiary hospitals, (Xi’an People’s Hospital (Xi’an Fourth Hospital) and the First Affiliated Hospital of Xi’an Jiaotong University), in Xi’an, China, between May 1, 2024, and January 31, 2026.

### 2.2 Study population

The inclusion criteria were as follows: (1) age over 18 years, (2) a documented clinical diagnosis of COPD and receipt of at least one inhaled medication prescribed specifically for COPD for 6 months or longer prior to hospitalization, and (3) sufficient cognitive ability to understand the purpose of the study and complete the questionnaires. Patients were enrolled based on the COPD diagnosis documented in their medical records. Pulmonary function results were available in the medical records for 416 participants. For the remaining 32 participants, pulmonary function result was unavailable in the medical records for this study; therefore, whether pulmonary function testing had been performed previously could not be verified. However, these participants had a clinical diagnosis of COPD documented in their medical records and were receiving inhaled treatment. Some patients were hospitalized because of exacerbation of respiratory symptoms, including shortness of breath, wheezing, chest tightness, cough, and sputum production, whereas others were admitted due to fever or secondary infection associated with an acute exacerbation of COPD. However, all participants were interviewed after clinical stabilization during hospitalization. All participants provided informed written consent to take part in the study.

The exclusion criteria were as follows: (1) inability to communicate due to language barrier, hearing impairment, visual impairment, psychological problems or mental illness; (2) history of tracheal intubation or mechanical ventilation due to acute COPD attack; (3) history of thoracic surgery or lung cancer; and (4) pregnancy.

### 2.3 Sample size

The required sample size for the study was calculated using the following formula: *n* = *z*^2^*p(1-p)/d*^2^, where *n* was the sample size, *z* was the standard normal deviate corresponding to a 95% confidence level (1.96), *p* was the proportion of patients with medication adherence, and *d* was the desired precision set at 0.05. Generally, adherence to long-term therapy for chronic diseases averages 50% (9). Based on these assumptions, a minimum sample size of 384 patients was required based on the above assumptions.

A total of 503 inpatients agreed to participate in the survey. Thirty-two inpatients were excluded based on predefined clinical criteria, and 23 questionnaires were incomplete. Finally, 448 (89.1%) valid responses were included in the analysis.

### 2.4 Survey procedures

In the inpatient department, potential participants were approached, informed about the study objectives, and assured of the anonymity and confidentiality of their responses. A face-to-face, interviewer-administered questionnaire was conducted by clinical pharmacists among the recruited inpatients with COPD. Demographic characteristics and instrument scores were collected by the investigators after obtaining informed consent. All investigators received standardized training to ensure objectivity and consistency during questionnaire administration. All participants were assessed at a fixed time on the day before discharge. A pilot study involving 40 participants was conducted first.

### 2.5 Measurement instruments

Two validated instruments developed by Professor Rob Horne were used in this study. The Medication Adherence Report Scale (MARS-5) was employed to evaluate adherence to inhaled medication, and the Beliefs about Medicines Questionnaire-Specific (BMQ-Specific) was used to assess patients’ medication beliefs. The Chinese versions of the MARS-5 and BMQ-Specific scales were adapted for use in our study after we obtained authorization from the developers of the scales (see Supplementary materials 1, 2).

#### 2.5.1 Medication adherence report scale (MARS-5)

The MARS-5 is a brief patient-reported measure of medication adherence that has demonstrated good psychometric properties in previous studies (22, 23). It comprises five items assessing both unintentional and intentional aspects of adherence behavior. Response options were scored on a five-point Likert scale, ranging from 1 (always) to 5 (never). Item scores are summed to obtain a total score ranging from 5 to 25, and higher scores indicate higher reported adherence to inhaled medications. According to previous literature (15, 24), a score of 25 was classified as perfect adherence, whereas scores <25 were classified as suboptimal adherence in the primary analysis. Furthermore, an alternative cut-off of 20 points was adopted in a sensitivity analysis, corresponding to 80% of the maximum possible score, with scores ≥20 classified as adherent and scores <20 classified as non-adherent (25).

Previous studies have used the 5-item MARS scale to access medication adherence among patients with COPD (11, 15, 24). The MARS-5 demonstrated modest internal consistency, with Cronbach’s α = 0.623 (95% CI = 0.557–0.679) and corrected item-total correlations (CITC) ranging from 0.377 to 0.460 in this study. Standardized factor loadings ranged from 0.610 to 0.901, indicating that all items loaded adequately onto the adherence factor (all>0.40).

#### 2.5.2 Beliefs about medicines questionnaire-specific (BMQ-specific)

The BMQ-Specific, which comprises two subscales (16, 17), was used to assess patients’ beliefs about inhaled medications. The scale contains 11 items, including five items assessing the perceived necessity of prescribed medication and six items assessing concerns about medication use. Responses are scored on a five-point Likert scale where 5 = strongly agree, 4 = agree, 3 = uncertain, 2 = disagree, and 1 = strongly disagree. Total scores range from 5 to 25 for the Necessity subscale and from 6 to 30 for the Concerns subscale. Higher scores on the subscales indicate stronger beliefs in the concepts they represent. Based on the use of the scale by Horne et al. (16), we used the midpoint as the cut-off between high and low beliefs (>17, ≤17 for the necessity beliefs about inhaled medications, and >15, ≤15 for the concerns beliefs about inhaled medications).

In previous studies, the BMQ-Specific was used to assess medication beliefs among patients with COPD (7, 11–13, 15, 26–28). The necessity subscale demonstrated good internal consistency, with a Cronbach’s α of 0.833 (95% CI = 0.805–0.856) and CITC values ranging from 0.600 to 0.704. The standardized factor loadings for the necessity items ranged from 0.788 to 0.932. In contrast, the concerns subscale demonstrated poor internal consistency, with a Cronbach’s α of 0.482 (95% CI = 0.407–0.546) and CITC values ranging from 0.020 to 0.429. The standardized factor loadings for the concerns items ranged from 0.103 to 0.691, and item C3 showed a particularly weak standardized factor loading (0.103). These results suggest that the necessity subscale has stronger psychometric support than the concerns subscale.

### 2.6 Data collection

Sociodemographic background (age, gender, smoking status, education, occupation, monthly income, place of residence) and variables related to disease and therapy (disease diagnosis, hospital admissions over the last year, comorbidities, and current use of prescription medication) were collected via face-to-face interviews with inpatients and further verified by reviewing medical records. Income is classified based on a defined threshold of 4,000 CNY per month: less than 4,000 CNY is considered low, and 4,000 CNY or more is considered high. Duration of inhaled medication use was confirmed via patient interview. The questionnaire adopted in our study was provided as a see Supplementary material 3. Description of current inhaled maintenance therapy covered by the survey was shown in Table 1.

**Table 1:** Current inhaled maintenance therapy covered by the survey.

Type of inhaled maintenance therapy	Specific agent
Single inhaler	
Triple therapy with LAMA-LABA-ICS	Budesonide, glycopyrronium bromide and formoterol fumarate inhalation aerosol (Breztri Aerosphere); Fluticasone furoate, umeclidinium bromide and vilanterol trifenatate powder for inhalation (Trelegy Ellipta)
Dual therapy with LABA-ICS	Salmeterol xinafoate and fluticasone propionate powder for inhalation (Seretide); Budesonide and formoterol fumarate powder for inhalation (Symbicort Turbuhaler)
Dual therapy with LAMA-LABA	Glycopyrronium bromide and formoterol fumarate inhalation aerosol (Bevespi Aerosphere); Indacaterol maleate and glycopyrronium bromide powder for inhalation (Ultibro Breezhaler); Umeclidinium bromide and vilanterol trifenatate powder for inhalation (Anoro Ellipta)
Monotherapy with LAMA	Tiotropium
Two separate inhalers	Salmeterol xinafoate and fluticasone propionate powder for inhalation (Seretide)/Budesonide and formoterol fumarate powder for inhalation (Symbicort Turbuhaler) + Tiotropium

LAMA, long-acting muscarinic antagonist; LABA, long-acting beta-agonist; ICS, inhaled corticosteroid.

### 2.7 Statistical analysis

Data from the survey were descriptively presented, where categorical variables were described using counts and percentages and non-normally distributed continuous variables as medians (interquartile ranges). Differences in the candidate variables between adherent and non-adherent groups were performed using the Chi-square test for categorical variables, the Mann–Whitney test for non-normally distributed continuous variables, and the independent samples t-test for normal continuous variables.

Univariable and multivariable logistic regression analyses were performed to examine factors associated with medication non-adherence. Because treatment regimen and number of inhaled medications used represented overlapping treatment characteristics, treatment regimen was retained in the adjusted model and number of inhaled medications used was excluded.

Three sensitivity analyses were conducted: (1) replacement of the dichotomized BMQ Necessity and Concerns variables with their continuous total scores; (2) exclusion of the 32 participants without an available pulmonary function result; (3) use of an alternative MARS-5 cut-off of 20 points, with scores <20 classified as non-adherent.

Receiver operating characteristic (ROC) analysis was conducted to assess the discriminative ability of the logistic regression model in identifying non-adherence to inhaled medication. The primary logistic regression model was internally validated using 1,000 bootstrap samples. Discrimination was assessed using the apparent AUC, optimism estimate, and optimism-corrected AUC, and calibration using the optimism-corrected intercept, slope, and calibration curve.

Analyses were performed using SPSS version 25.0 Statistical Software Package for Windows and R version 4.6.1. The adjusted logistic regression models, sensitivity analyses, and bootstrap internal validation were performed in R. A *p* value<0.05 was considered statistically significant.

## 3 Results

### 3.1 General characteristics of participants

We recruited 448 inpatients, with a mean age of 68.8 years (SD = 9.5). Based on the self-reported medication adherence scale, 182 (40.6%) inpatients were adherent to inhaled medication in this study. The majority of participants (74.1%) were male. Approximately 95.3% of inpatients were prescribed a single inhaler, while the remaining 4.7% were prescribed two separate inhalers. Among the inpatients, 61.8% used the inhaler once a day, while the remainder used it twice a day. Regarding the type of inhaled medication prescribed, the most common maintenance regimens were dual therapy (LABA-LAMA: 48.2% and LABA-ICS: 22.8%), followed by triple therapy with LAMA-LABA-ICS (18.8%), and 19 (4.2%) participants used two separate inhalers. The mean score on the MARS among the respondents was 22.84 (SD = 2.51). Of the 448 inpatients, 243 (54.2%) expressed strong necessity beliefs about inhalers. In contrast, 203 (45.3%) held strong concern beliefs about inhalers. The mean score was 17.1 (SD = 3.1) for necessity beliefs and 15.6 (SD = 2.6) for concern beliefs. Table 2 presents the detailed demographic and clinical characteristics of the study participants.

**Table 2:** Demographic and clinical characteristics of the study subjects.

Characteristics	Total (*n* = 448, %)	Adherent (*n* = 182, %)	Non-adherent (*n* = 266, %)	*p*-value
Age, Median (IQR)	69.0 (62.8, 74.2)	69.0 (62.0, 74.8)	69.0 (63.0, 74.0)	0.946
Age (years)				0.759
<65	150(33.5)	64(35.2)	86(32.3)	
65–74	186(41.5)	72(39.6)	114(42.9)	
≥75	112(25.0)	46(25.3)	66(24.8)	
Gender				0.493
Female	116(25.9)	44(24.2)	72(27.1)	
Male	332(74.1)	138(75.8)	194(72.9)	
Smoking status				0.205
Never smoked before	225(50.2)	87(47.8)	138(51.9)	
Current smoker	105(23.4)	39(21.4)	66(24.8)	
Ex-smoker	118(26.3)	56(30.8)	62(23.3)	
Education level				**0.002**
≤High school	353(78.8)	130(71.4)	223(83.8)	
>High school	95(21.2)	52(28.6)	43(16.2)	
Occupation				**<0.001**
Physical labor	166(37.1)	48(26.4)	118(44.4)	
Office work	58(12.9)	30(16.5)	28(10.5)	
Unemployed/Retired	224(50.0)	104(57.1)	120(45.1)	
Income				**0.002**
Low	308(68.8)	110(60.4)	198(74.4)	
High	140(31.2)	72(39.6)	68(25.6)	
Place of residence				**0.422**
Urban	293(65.4)	123(67.6)	170(63.9)	
Rural	155(34.6)	59(32.4)	96(36.1)	
The number of hospital admissions due to exacerbations over the last year				0.992
0	112(25.0)	46(25.3)	66(24.8)	
Once	239(53.3)	97(53.3)	142(53.4)	
≥Twice	97(21.7)	39(21.4)	58(21.8)	
Comorbidities				
Heart and cardiovascular disease	142(31.7)	61(33.5)	81(30.5)	0.493
Diabetes	77(17.2)	26(14.3)	51(19.2)	0.178
Hyperlipidemia	17(3.8)	7(3.8)	10(3.8)	0.962
Hypertension	190(42.4)	84(46.2)	106(39.8)	0.185
Asthma	34(7.6)	19(10.4)	15(5.6)	0.060
Duration of inhaled medication use (years)				0.153
<1	145(32.4)	56(30.8)	89(33.5)	
1–5	203(45.3)	77(42.3)	126(47.4)	
>5	100(22.3)	49(26.9)	51(19.1)	
Type of inhaled maintenance therapy				
Single inhaler				
Triple therapy with LAMA-LABA-ICS	84(18.8)	45(24.7)	39(14.7)	0.092
Dual therapy with LABA-ICS	102(22.8)	39(21.4)	63(23.7)	
Dual therapy with LAMA-LABA	216(48.2)	83(45.6)	133(50.0)	
Monotherapy with LAMA	27(6.0)	8(4.4)	19(7.1)	
Two separate inhalers	19(4.2)	7(3.8)	12(4.5)	
Number of inhaled medications used				0.809
1	427(95.3)	174(95.6)	253(95.1)	
2	21(4.7)	8(4.4)	13(4.9)	
Daily dosing frequency for inhaled medication				0.273
Once	277(61.8)	107(58.8)	170(63.9)	
Twice	171(38.2)	75(41.2)	96(36.1)	
Medications prescribed for other chronic diseases				0.918
Yes	235(52.5)	96(52.7)	139(52.3)	
No	213(47.5)	86(47.3)	127(47.7)	
Necessity beliefs (mean score ± SD)	17.09 ± 3.10	18.43 ± 2.14	16.17 ± 3.32	**<0.001**
Concerns beliefs (mean score ± SD)	15.64 ± 2.56	15.48 ± 2.54	15.75 ± 2.58	0.276

Bold values indicated a *p*-value< 0.05. Continuous variables are presented as mean ± SD or median (IQR), and categorical variables as n (%).

### 3.2 Logistic regression analysis for non-adherence to inhaled medication

Table 3 presents the results of the univariable and multivariable logistic regression analyses of factors associated with non-adherence to inhaled medication.

**Table 3:** Univariable and multivariable logistic regression analyses of factors associated with non-adherence to inhaled medication.

Characteristics	Unadjusted OR (95% CI)	*p-*value	Adjusted OR (95% CI)	*p-*value
Age (years)				
<65	1.000(Reference)		1.000(Reference)	
65–74	1.178 (0.760, 1.826)	0.463	1.121 (0.681, 1.848)	0.653
≥75	1.068 (0.650, 1.754)	0.796	1.375 (0.755, 2.501)	0.298
Gender				
Female	1.000(Reference)		1.000(Reference)	
Male	0.859 (0.557, 1.326)	0.493	1.223 (0.670, 2.232)	0.512
Smoking status				
Never smoked before	1.000(Reference)		1.000(Reference)	
Current smoker	1.067 (0.661, 1.721)	0.791	1.314 (0.709, 2.433)	0.386
Ex-smokers	0.698 (0.445, 1.095)	0.117	0.640 (0.355, 1.152)	0.137
Education level				
≤High school	1.000(Reference)		1.000(Reference)	
>High school	0.482 (0.305, 0.762)	**0.002**	0.865 (0.434, 1.723)	0.680
Occupation				
Physical labor	1.000(Reference)		1.000(Reference)	
Office work	0.380 (0.205, 0.702)	**0.002**	0.628 (0.269, 1.469)	0.283
Unemployed/Retired	0.469 (0.307, 0.719)	**0.001**	0.534 (0.320, 0.889)	**0.016**
Income				
Low	1.000(Reference)		1.000(Reference)	
High	0.525 (0.350, 0.787)	**0.002**	0.578 (0.305, 1.098)	0.094
Place of residence				
Urban	1.000(Reference)		1.000(Reference)	
Rural	1.177 (0.790, 1.754)	0.422	0.969 (0.596, 1.576)	0.898
The number of hospital admissions due to exacerbations over the last year				
0	1.000(Reference)		1.000(Reference)	
Once	1.020 (0.646, 1.611)	0.931	0.871 (0.516, 1.469)	0.604
≥Twice	1.037 (0.596, 1.803)	0.899	0.879 (0.460, 1.681)	0.697
Duration of inhaled medication use (years)				
<1	1.000(Reference)		1.000(Reference)	
1–5	1.030 (0.664, 1.596)	0.896	1.369 (0.828, 2.264)	0.221
>5	0.655 (0.391, 1.096)	0.107	1.090 (0.583, 2.038)	0.787
Type of inhaled maintenance therapy				
Triple therapy with LAMA-LABA-ICS	1.000(Reference)		1.000(Reference)	
Dual therapy with LABA-ICS	1.864 (1.037, 3.349)	**0.037**	2.001 (1.027, 3.900)	**0.042**
Dual therapy with LAMA-LABA	1.849 (1.111, 3.076)	**0.018**	1.613 (0.774, 3.362)	0.202
Monotherapy with LAMA	2.740 (1.080, 6.950)	**0.034**	1.787 (0.556, 5.750)	0.330
Two separate inhalers	1.978 (0.709, 5.519)	0.193	2.695 (0.826, 8.793)	0.100
Daily dosing frequency for inhaled medication				
Once	1.000(Reference)		1.000(Reference)	
Twice	0.806 (0.547, 1.186)	0.274	0.816 (0.415, 1.602)	0.554
Medications prescribed for other chronic diseases				
No	1.000(Reference)		1.000(Reference)	
Yes	0.980 (0.672, 1.430)	0.918	0.907 (0.587, 1.402)	0.660
Necessity beliefs				
High	1.000(Reference)		1.000(Reference)	
Low	4.202 (2.782, 6.347)	**<0.001**	4.785 (3.044, 7.520)	**<0.001**
Concerns beliefs				
High	1.000(Reference)		1.000(Reference)	
Low	0.756(0.516, 1.106)	0.149	0.683(0.439, 1.062)	0.091

Bold values indicated a *p*-value < 0.05. OR, odds ratio; CI, confidence interval.

According to the univariable analysis, inpatients with the following characteristics were more likely to be adherent to inhaled medication: higher income (unadjusted odds ratio [OR] and 95% confidence interval [CI] 0.525 [0.350, 0.787]; *p* = 0.002), higher educational level (unadjusted OR 0.482 [0.305, 0.762]; *p* = 0.002), office work (unadjusted OR 0.380 [0.205, 0.702]; *p* = 0.002), and unemployment or retirement (unadjusted OR 0.469 [0.307, 0.719]; *p* < 0.001). Inpatients with weak necessity beliefs about their inhaled medication were more likely to be non-adherent (unadjusted OR 4.202 [2.782, 6.347], *p* < 0.001). Inpatient who received dual therapy with LABA-ICS (unadjusted OR 1.864 [1.037, 3.349]; *p* = 0.037) and LABA-LAMA (unadjusted OR 1.849 [1.111, 3.076]; *p* = 0.018), or monotherapy with LAMA (tiotropium) inhaler (unadjusted OR 2.740 [1.080, 6.950]; *p* = 0.034), were more likely to be non-adherent than those who received triple therapy with LABA-LAMA-ICS.

Multivariable logistic regression analysis revealed that inpatient who expressed weaker necessity beliefs about inhaled medication had 4.79-fold odds (95% CI = 3.044–7.520; *p* < 0.001) of non-adherence compared with those who expressed stronger necessity beliefs. Inpatient who received dual therapy with LABA-ICS had 2.00-fold odds (95% CI = 1.027–3.900; *p* = 0.042) of non-adherence compared with those who received triple therapy with LAMA-LABA-ICS, whereas inpatient who was unemployed or retired was more likely to be adherent to inhaled medication (adjusted OR 0.534 [0.320, 0.889]; *p* = 0.016) than those in physical labor occupations.

### 3.3 Sensitivity analyses

Three sensitivity analyses were provided as a Supplementary material 4. In this multicenter cross-sectional study of inpatients with COPD, we found that weaker necessity beliefs about inhaled medications were associated with medication non-adherence. This association remained statistically significant after modeling BMQ Necessity and Concerns scores as a continuous variable, excluding participants without available pulmonary function results, and applying an alternative MARS-5 cut-off (Supplementary Table S1). When BMQ-Specific subscale scores were analyzed as continuous variables, inpatient who expressed stronger necessity beliefs about inhaled medication was associated with lower odds of non-adherence (adjusted OR 0.723 [0.659, 0.793]; *p* < 0.001). Inpatient who received dual therapy with LABA-ICS (adjusted OR 2.110 [1.073, 4.147]; *p* = 0.030) were significantly associated with non-adherence. Inpatient who was unemployed or retired (adjusted OR 0.549 [0.328, 0.919]; *p* = 0.023) was significantly associated with adherence (Supplementary Table S2).

After excluding the 32 participants whose pulmonary function result was unavailable in the medical records, 416 participants remained. Weaker necessity beliefs remained significantly associated with non-adherence (adjusted OR 4.916 [3.082, 7.842]; *p* < 0.001). High income level was significantly associated with medication adherence (adjusted OR 0.473 [0.242, 0.926]; *p* = 0.029). However, the associations between unemployment or retirement (adjusted OR 0.608 [0.358, 1.032]; *p* = 0.065), LABA-ICS therapy (adjusted OR 1.981 [0.987, 3.978]; *p* = 0.055) and medication adherence were attenuated and no longer statistically significant (Supplementary Table S3).

When the alternative MARS-5 cut-off of 20 points was applied, 51 (11.4%) participants were classified as non-adherent. Weaker necessity beliefs remained significantly associated with non-adherence (adjusted OR 13.402 [5.373, 33.430]; *p* < 0.001). The associations between unemployment or retirement (adjusted OR 1.517 [0.719, 3.202]; *p* = 0.274), LABA-ICS therapy (adjusted OR 2.348 [0.706, 7.806]; *p* = 0.164) and medication adherence were no longer statistically significant using this alternative MARS-5 cut-off of 20 points, and estimates for some covariates had wider confidence intervals because of the smaller number of non-adherent participants (Supplementary Table S4).

### 3.4 Internal validation

Figure 1 shows the apparent receiver operating characteristic (ROC) curve for the logistic regression model of non-adherence to inhaled medication. The apparent area under the curve (AUC) was 0.752 (95% CI = 0.708–0.796).

**Figure 1:**
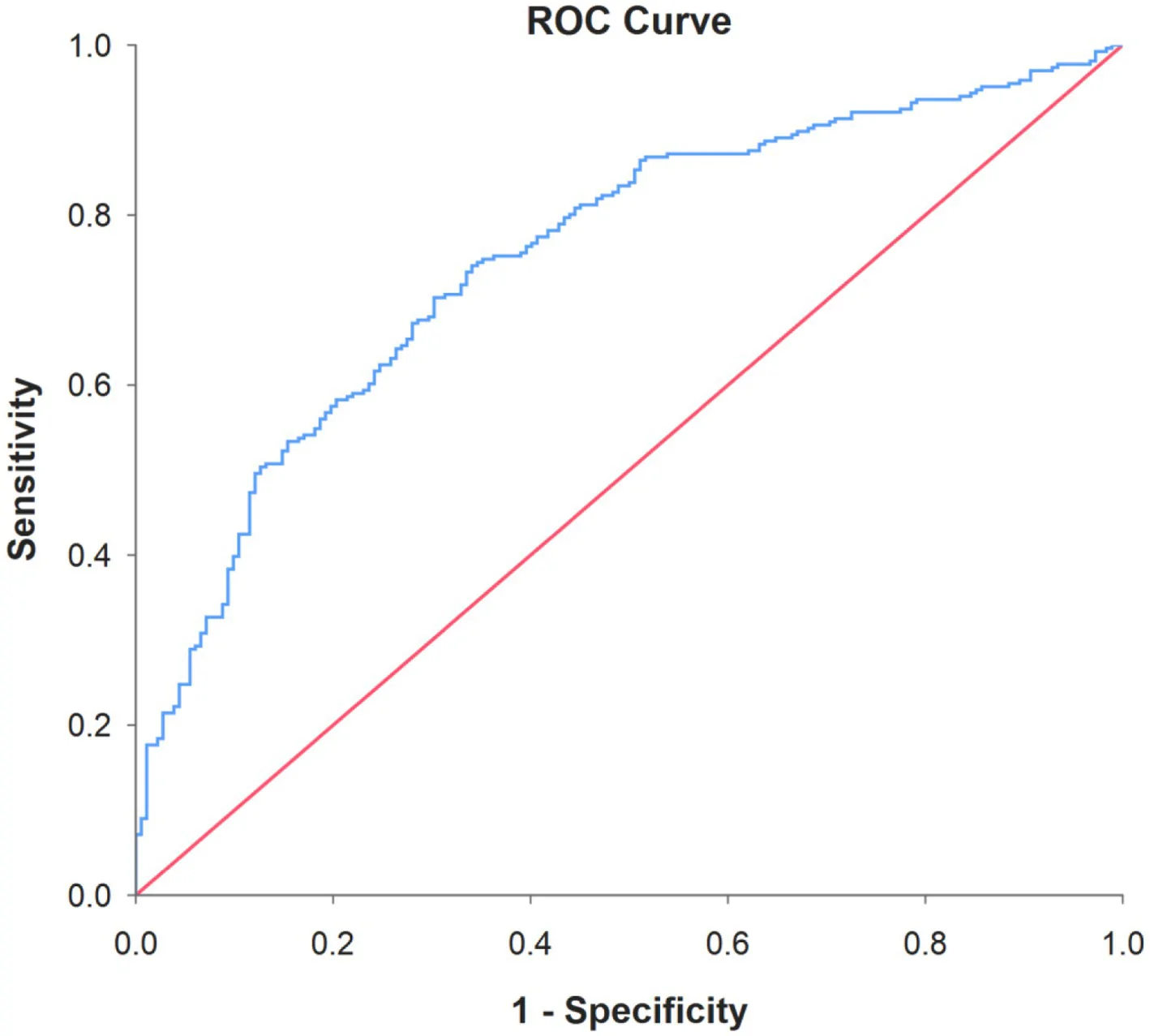
Receiver operating characteristic (ROC) curve for the primary logistic regression model of non-adherence to inhaled medication. The apparent AUC was 0.752 (95% CI 0.708–0.796).

Internal validation using 1,000 bootstrap samples yielded an apparent AUC of 0.752, mean optimism of 0.052, and optimism-corrected AUC of 0.700. The optimism-corrected calibration intercept and slope were −0.007 and 0.740, respectively, indicating some optimism in the apparent model performance (Figure 2).

**Figure 2:**
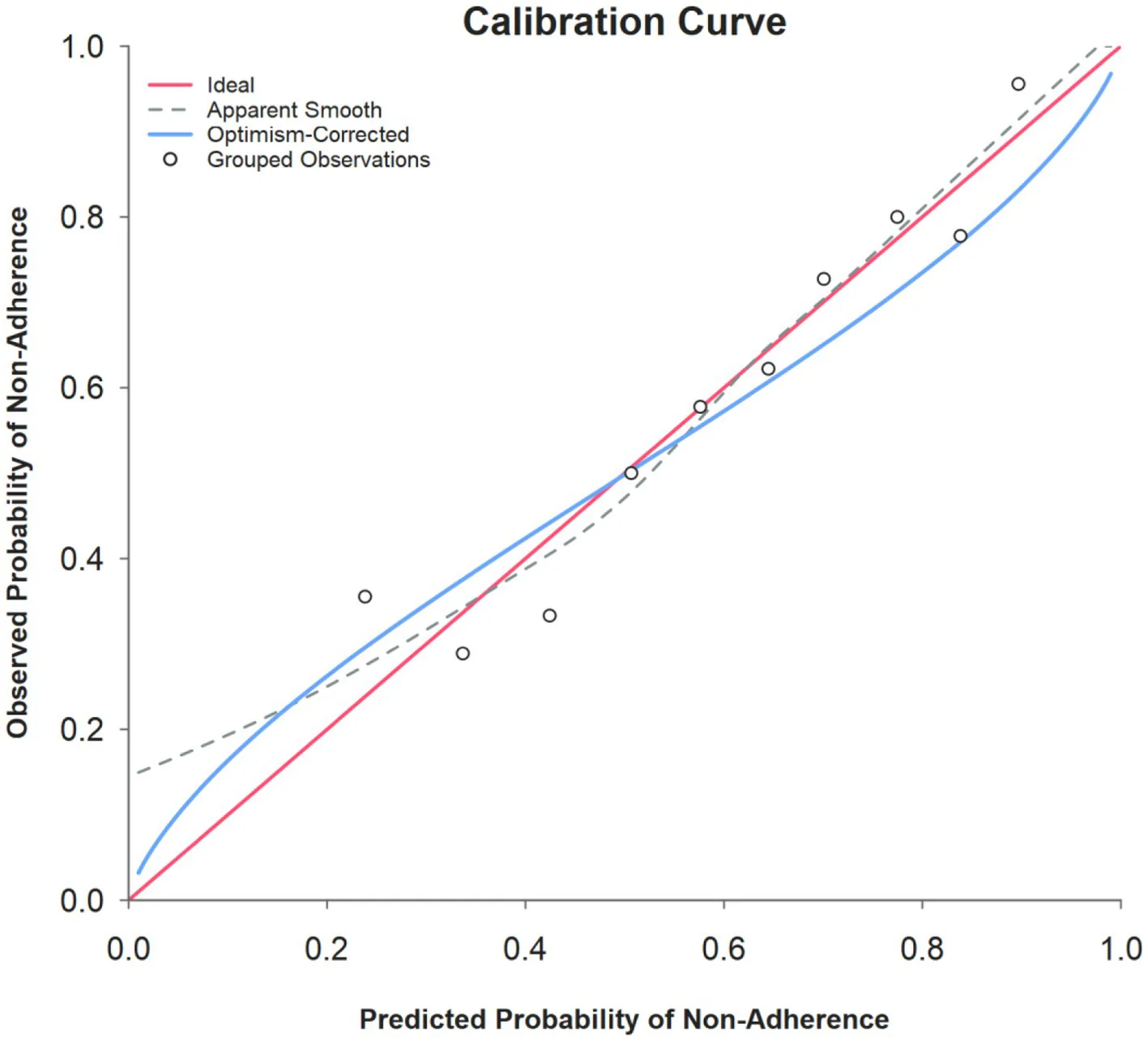
Bootstrap-corrected calibration curve for the logistic regression model assessing non-adherence to inhaled medication. The apparent and optimism-corrected calibration curves are shown together with grouped observations and the ideal 45-degree calibration line. Internal validation was performed using 1,000 bootstrap resamples.

## 4 Discussion

In this study, a total of 182 (40.6%) inpatients with COPD were adherent to their inhalers. According to the Global Initiative for Chronic Obstructive Lung Disease (2023), adherence to inhalation therapy is below 50% (29). The prevalence of medication adherence in the current study was higher than that reported in previous findings in China [7.5% (7), 15.7% (13), 37.8% (30)], as well as in other countries such as Poland [26% (12)], Germany [34% (15)], and Australia [33.6% (14), 37% (24)], but lower than those reported in the USA [42% (27)] and Portugal [83.3% (26)]. Over a 6-month follow-up period, a multicenter prospective observational study in China found that 66.8% of COPD patients adhered to their prescribed medications, whereas a study in Spain reported that only approximately 40% of newly treated patients continued with their inhalation therapy (31, 32). Another study conducted in Germany reported that 43% of patients with COPD were adherent at the 3-month follow-up, compared with 46% at the 1-year follow-up (11). This discrepancy might be explained by differences in adherence measurement tools and baseline patient characteristics across studies.

We found that necessity beliefs about inhalers were associated with medication adherence in inpatients with COPD, which was consistent with previous findings (12, 15, 26). However, no correlation was found between concern beliefs about inhalers and medication adherence in this study. A possible explanation is that patients may develop stronger beliefs in the necessity of medication while their concerns are reduced, and this belief-concern discrepancy is associated with medication adherence (7). Additionally, concerns about inhaled medications did not appear to be significantly associated with medication adherence in COPD, likely due to the low incidence of side effects perceived by patients (26). However, it was also reported that necessity beliefs about medication were positively associated with adherence, while concern beliefs were negatively associated with adherence among patients with COPD (7, 11, 13), which was inconsistent with our study. In a study in the Netherlands, not only self-reported adherence but also adherence assessed via prescription-refill records correlated with patients’ beliefs about inhaled corticosteroids (ICS) - namely, necessity and concerns (33). In a cohort of urban minority adults in the United States, only concern beliefs about medications were associated with non-adherence (27), which was inconsistent with our findings. Future studies should involve larger and more geographically diverse samples to confirm the broader applicability of the findings. Our findings provide insights for healthcare providers to examine medication beliefs during patient visits and to provide counseling to rectify misperceptions and erroneous beliefs, which was consistent with previous findings (28). As an important and modifiable aspect of healthcare, patient-doctor communication should be improved when prescribing inhalers for individuals with COPD (15). This involves providing accurate information and educational interventions to enhance patients’ medication beliefs.

In previous studies, little is known about the correlation between type of inhaled maintenance therapy and medication adherence among patients with COPD. Maintenance inhalers can be given as monotherapy, which can be escalated to dual or triple therapy if needed (e.g., LABA-ICS or LAMA-LABA-ICS) (34). We found that inpatient who received the triple therapy regimen (LAMA-LABA-ICS) were more likely to be adherent compared with those who received the dual therapy regimen (LABA-ICS). COPD patients who received the triple therapy regimen (LAMA-LABA-ICS) may have more severe conditions. Given the significant symptom burden and risks associated with more severe disease, it would seem logical that these patients would have a greater incentive to adhere to prescribed therapy, and hence a greater likelihood of adherence (9, 14). Studies confirm that increased adherence is associated with the level of symptom control (12). Another possible explanation is that the superior clinical efficacy of triple therapy (LAMA-LABA-ICS) over dual therapy (LABA-ICS) may be associated with better adherence, which is consistent with previous research (35). It was reported that the triple therapy regimen (LAMA-LABA-ICS) offered greater advantages over the dual therapy (LABA-ICS) in reducing acute exacerbations, improving lung function, and lowering all-cause mortality (36, 37). GOLD 2023 explicitly advises against the use of LABA-ICS in COPD patients without concomitant asthma (38). The use of inhaled LABA-ICS has been progressively restricted. It recommends LAMA-LABA as the initial therapy for Groups B and E, or LAMA-LABA-ICS for Group E patients with blood eosinophil counts ≥300 cells/μL (38). Despite the progressive restriction of LABA-ICS in GOLD 2023, earlier prescribing habits among primary care providers may have contributed to the 22.8% prescription rate for LABA-ICS among inpatients in our study (conducted during 2024–2026) (39). Future studies should consider stratifying by GOLD A/B/E clinical grouping when examining the associations between inhaled treatment regimens and medication adherence among patients with COPD.

In accordance with previous studies (12), we found that unemployment or retirement was positively associated with medication adherence in patients with COPD. One possible reason is that unemployed or retired patients have more leisure time, allowing them to devote more attention to medication adherence, clinic visits, and breathing exercises (12). Further research is needed to confirm the broader applicability of the findings.

In the univariable analysis, our study demonstrated that higher educational levels and higher monthly income were significantly associated with better adherence to inhaled medications among inpatients with COPD. However, through multivariable logistic regression analysis, educational level and income level were not found to be significantly associated with adherence to medications. Previous research emphasized that education level and monthly income are associated with chronic disease self-management (30, 40). It was reported that COPD patients with inadequate health literacy exhibited poorer self-management behaviors, including lower medication adherence and incorrect inhaler use (41). Our findings were inconsistent with previous studies (42). The possible reasons are as follows. The sample of this study was limited to inpatients from two tertiary hospitals in Xi’an City, which may not adequately represent the diverse COPD patient population in China and may introduce selection bias. Future research should expand the geographic scope of participant recruitment to strengthen the representativeness and generalizability of the findings. The number of hospital admissions due to exacerbations of the disease over the last year was not found to be an independent determinant of medication non-adherence, which was inconsistent with previous study (12). Further research is needed to comprehensively explore the association between demographic and clinical characteristics and medication adherence among COPD patients.

Self-management behaviors in COPD patients were suboptimal (43). COPD necessitates long-term inhaler therapy, which poses several challenges for patients, including the need to master different inhalation devices (each with its own technique), the risk of inhaler usage errors, and the burden of complex dosing regimens (44). Previous studies have shown that it is difficult for patients to master the operation steps of inhaler devices (8). Older adults are more prone to the misuse of inhaler devices, which could lead to poor therapeutic outcomes and disease control (45). “I vary my recommended management based on how I am feeling” and “I get confused about my medications” were found to be significant associated with non-adherence in the Australian COPD population (24). As recommended by previous studies, enhancing patients’ understanding of proper inhaler use and its long-term benefits may improve the quality of life in COPD patients (46, 47). Effective communication between healthcare providers and patients might be one of the simplest and most cost-effective strategies for improving adherence (9, 43, 48). Multidimensional pharmaceutical care, including inhalation technique training and education, was effective in improving and maintaining proper inhaler technique among COPD patients (8, 49). Furthermore, nurses can also provide accurate information and expertise related to inhaler technique (50). Existing studies have shown that interventions by pharmacists and nurses can improve medication adherence or clinical outcomes in patients with COPD (51–55). More intervention approaches, including WeChat-based programs, pictorial action plans (PAP), and virtual reality, could be incorporated into chronic disease management (56–59).

### 4.1 Strengths and limitations

This study is the first to explore the associations between medication beliefs and adherence to inhaled medication among patients with COPD in Xi’an, China. Our findings provide insights for healthcare providers to design tailored strategies to improve adherence among patients with COPD. Our study has exhibited several limitations. First, because our cohort was recruited from inpatient wards of two hospitals in a single urban area in China, the study results were limited by the relatively homogeneous sample. Given the limitations of the current sample, future studies with larger samples and more sociodemographically diverse populations are needed to validate the robustness of our findings. Second, the association between treatment regimen and adherence may be confounded by disease severity, symptoms, GOLD classification, inhaler technique, health literacy and cognitive status. We did not analyze adherence in patients stratified by disease severity or GOLD A/B/E groups. Disease severity was associated with inhaler type and adherence. However, it should be noted that pulmonary function results were unavailable in the medical records for 32 participants. The absence of these data made it difficult to grade disease severity in these patients. COPD-specific symptom scores [modified Medical Research Council (mMRC) and COPD Assessment Test (CAT)] were not obtained in the cohort study. Participants were not stratified into GOLD groups A, B and E using baseline CAT score and exacerbation history in our study. This approach might have introduced bias, potentially affecting the generalizability of our findings. Third, the BMQ-Specific concerns subscale demonstrated poor internal consistency and weak item-level performance in this sample. Therefore, the absence of an association between concerns and adherence should be interpreted cautiously. Additionally, adherence and medication beliefs were assessed using self-reported instruments, which may introduce recall or social desirability bias. The MARS-5 also demonstrated modest internal consistency in this sample, which may have introduced measurement error in the assessment of adherence.

## 5 Conclusion

Adherence rates to inhaled medication have been found to be low among inpatients with COPD in Xi’an. We found that necessity beliefs about inhaled medication were associated with medication adherence in inpatients with COPD. These findings suggest a potential clinical implication wherein pharmacists and nurses could play a supportive role in promoting medication adherence among COPD patients.

## Data Availability

The original contributions presented in the study are included in the article/Supplementary material, further inquiries can be directed to the corresponding authors.
