# Supplementary material for: Associations between medication beliefs and medication adherence among inpatients with chronic obstructive pulmonary disease: a cross-sectional survey
Source: Front Public Health. 2026 Sep 16;14:1934660. doi: 10.3389/fpubh.2026.1934660 (PMC13624166; doi:10.3389/fpubh.2026.1934660)
Supplement: Supplementary file 1 [file Data_Sheet_1.PDF]

QUESTIONS ABOUT USING YOUR MEDICINES

- **Many people find a way of using their medicines which suits them.**
- **This may differ from the instructions on the label or from what their doctor has said.**
- **We would like to ask you a few questions about how you use your medicines**

Here are some ways in which people have said that they use their medicines

For each of the statements, please tick the box which best applies to you

	Your own way of using your medicines	Always	Often	Sometimes	Rarely	Never
M1	I forget to take them					
M2	I alter the dose					
M3	I stop taking them for a while					
M4	I decide to miss out a dose					
M5	I take less than instructed					

 © Professor Rob Horne
