# Supplementary material for: Associations between medication beliefs and medication adherence among inpatients with chronic obstructive pulmonary disease: a cross-sectional survey
Source: Front Public Health. 2026 Sep 16;14:1934660. doi: 10.3389/fpubh.2026.1934660 (PMC13624166; doi:10.3389/fpubh.2026.1934660)
Supplement: Supplementary file 2 [file Data_Sheet_2.PDF]

YOUR VIEWS ABOUT

MEDICINES PRESCRIBED FOR YOU

(BMQ SPECIFIC)

We would like to ask you about your personal views about medicines prescribed for you. These are statements other people have made about their medicines.
Please show how much you agree or disagree with them by ticking the appropriate box.

**There are no right or wrong answers.
We are interested in your personal views**

	Views about MEDICINES PRESCRIBED FOR YOU:	Scale Structure
BS1	My health, at present, depends on my medicines N	N= Specific Necessity
BS2	Having to take medicines worries me C	C= Specific Concerns
BS3	My life would be impossible without my medicines N	
BS5	I sometimes worry about long-term effects of my medicines C	
BS4	Without my medicines I would be very ill N	
BS6	My medicines are a mystery to me C	
BS7	My health in the future will depend on my medicines N	
BS8	My medicines disrupt my life C	
BS9	I sometimes worry about becoming too dependent on my medicines C	
BS10	My medicines protect me from becoming worse N	
BS11	These medicine give me unpleasant side effects C	

All items scored: 5= strongly agree, 4= agree, 3= uncertain, 2= disagree, 1= strongly disagree

FORMULA FOR COMPUTING BMQ SPECIFIC SCALES

5 item Sepcific Necessity scale

- sum of scores on all N items. Divide by 5 (number of items in scale) to give a scale score (range 1-5)

6-item Specific Concerns

- sum of scores on all N items. Divide by 6 (number of items in scale) to give a scale score (range 1-5)

SCORING INSTRUCTIONS BMQ-Specific 11 _04 (3) © Professor Rob Horne 1999
