# Supplementary material for: Associations between medication beliefs and medication adherence among inpatients with chronic obstructive pulmonary disease: a cross-sectional survey
Source: Front Public Health. 2026 Sep 16;14:1934660. doi: 10.3389/fpubh.2026.1934660 (PMC13624166; doi:10.3389/fpubh.2026.1934660)
Supplement: Supplementary file 3 [file Data_Sheet_3.PDF]

Hello! These are statements we have made about the inhaled medication prescribed for you. Please truthfully fill in the following information and tick the appropriate option that best suits your situation. There are no right or wrong answers. We are interested in your personal views. Your answer will be kept confidential and will not have any impact on your treatment.

Age (years):

Gender: A. Female B. male

Smoking status:

A. Never smoked before B. Current smoker C. Ex-smoker

Education level: A. ≤ High school

B. >High school

Occupation: A. Physical labor B. Office work C. Unemployed/Retired

Income/month: A. <4000 yuan B. ≥4000 yuan

Place of residence: A. Urban B. Rural

The number of hospital admissions due to exacerbations over the last year?

A. 0 B. Once C. ≥2 times

How long have you used the inhaled medications?

A. <1 years B. 1-4 years C. ≥5 years

Among the following medications, please check the inhaled medications you are currently using:

A. Budesonide, glycopyrronium bromide and formoterol fumarate inhalation aerosol (Breztri Aerosphere)
B. Fluticasone furoate, umeclidinium bromide and vilanterol trifenatate powder for inhalation (Trelegy Ellipta)
C. Salmeterol xinafoate and fluticasone propionate powder for inhalation (Seretide)
D. Budesonide and formoterol fumarate powder for inhalation (Symbicort Turbuhaler)
E. Glycopyrronium bromide and formoterol fumarate inhalation aerosol (Bevespi Aerosphere)

F. Indacaterol maleate and glycopyrronium bromide powder for inhalation (Ultibro Breezhaler)
G. Umeclidinium bromide and vilanterol trifenatate powder for inhalation (Anoro Ellipta)
H. Tiotropium
I. Salmeterol xinafoate and fluticasone propionate powder for inhalation (Seretide) + Tiotropium
J. Budesonide and formoterol fumarate powder for inhalation (Symbicort Turbuhaler) + Tiotropium

Number of inhaled medications used: A. One B. Two

Daily dosing frequency for inhaled medication: A. Once B. Twice

Medications prescribed for other chronic diseases: A. No B. Yes

Medication Adherence Report Scale (MARS-5)

Many people find a way of using their medicines which suits them. This may differ from the instructions on the label or from what their doctor has said. We would like to ask you a few questions about how you use your inhaled medicines. For each of the statements, please tick the answer which best applies to you.

1. I forget to take them

   A. Always B. Often C. Sometimes D. Rarely E. Never

2. I alter the dose

   A. Always B. Often C. Sometimes D. Rarely E. Never

3. I stop taking them for a while

   A. Always B. Often C. Sometimes D. Rarely E. Never

4. I decide to miss out a dose

A. Always B. Often C. Sometimes D. Rarely E. Never

5. I take less than instructed

   A. Always B. Often C. Sometimes D. Rarely E. Never

Beliefs about Medicines Questionnaire-Specific (BMQ-Specific)

We would like to ask you about your personal views about the inhaled medicines prescribed for you. Please show how much you agree or disagree with them by ticking the appropriate option. There are no right or wrong answers. We are interested in your personal views.

1. My health, at present, depends on my medicines.

   A. strongly agree B. agree C. uncertain D. disagree E. strongly disagree

2. Having to take medicines worries me.

   A. strongly agree B. agree C. uncertain D. disagree E. strongly disagree

3. My life would be impossible without my medicines.

   A. strongly agree B. agree C. uncertain D. disagree E. strongly disagree

4. I sometimes worry about long-term effects of my medicines.

   A. strongly agree B. agree C. uncertain D. disagree E. strongly disagree

5. Without my medicines I would be very ill.

   A. strongly agree B. agree C. uncertain D. disagree E. strongly disagree

6. My medicines are a mystery to me.

   A. strongly agree B. agree C. uncertain D. disagree E. strongly disagree

7. My health in the future will depend on my medicines.

   A. strongly agree B. agree C. uncertain D. disagree E. strongly disagree

8. My medicines disrupt my life.

   A. strongly agree B. agree C. uncertain D. disagree E. strongly disagree

9. I sometimes worry about becoming too dependent on my medicines.

   A. strongly agree B. agree C. uncertain D. disagree E. strongly disagree

10. My medicines protect me from becoming worse.

    A. strongly agree B. agree C. uncertain D. disagree E. strongly disagree

11. These medicine give me unpleasant side effects.

    A. strongly agree B. agree C. uncertain D. disagree E. strongly disagree
