# Supplementary material for: Associations between medication beliefs and medication adherence among inpatients with chronic obstructive pulmonary disease: a cross-sectional survey
Source: Front Public Health. 2026 Sep 16;14:1934660. doi: 10.3389/fpubh.2026.1934660 (PMC13624166; doi:10.3389/fpubh.2026.1934660)
Supplement: Supplementary file 4 [file Data_Sheet_4.PDF]

Supplementary Table S1 Overview of primary and sensitivity analyses of factors associated with non-adherence to inhaled medication

Analysis	MARS-5 cut-off	N/non-adherent (%)	The adjusted logistic regression of factors associated with non-adherence to inhaled medication
**Primary**	<25	448/266 (59.4%)	Inpatient who expressed weaker necessity beliefs about inhaled medication and received dual therapy with LABA-ICS were significantly associated with non-adherence. Inpatient who was unemployed or retired was significantly associated with adherence.
**Continuous BMQ-Specific scores**	<25	448/266 (59.4%)	Inpatient who expressed stronger necessity beliefs about inhaled medication was associated with lower odds of non-adherence. Inpatient who received dual therapy with LABA-ICS was significantly associated with non-adherence. Inpatient who were unemployed or retired was significantly associated with adherence.
**Exclusion of the 32 participants without an available pulmonary function result**	<25	416/242 (58.2%)	Inpatient who expressed weaker necessity beliefs about inhaled medication was significantly associated with non-adherence. High income level was significantly associated with medication adherence.
**Alternative MARS-5 cut-off**	<20	448/51 (11.4%)	Inpatient who expressed weaker necessity beliefs about inhaled medication was significantly associated with non-adherence.

Supplementary Table S2 Multivariable logistic regression analyses of factors associated with non-adherence to inhaled medication (Adjusted model using continuous BMQ-Specific scores)

Characteristics	Adjusted OR (95% CI)	*P-value*
**Age (years)**		
<65	1.000(Reference)	
65-74	1.089 (0.657-1.805)	0.742
≥75	1.428 (0.776-2.627)	0.253
**Gender**		
Female	1.000(Reference)	
Male	1.247 (0.676-2.300)	0.479
**Smoking status**		
Never smoked before	1.000(Reference)	
Current smoker	1.342 (0.718-2.509)	0.356
Ex-smokers	0.620 (0.342-1.126)	0.116
**Education level**		
≤High school	1.000(Reference)	
>High school	0.815 (0.406-1.638)	0.566
**Occupation**		
Physical labor	1.000(Reference)	
Office work	0.582 (0.248-1.369)	0.215
Unemployed/Retired	0.549 (0.328-0.919)	0.023
**Income**		
Low	1.000(Reference)	
High	0.664 (0.348-1.264)	0.213
**Place of residence**		
Urban	1.000(Reference)	
Rural	1.134 (0.693-1.855)	0.617
**The number of hospital admissions due to exacerbations over the last year**		
0	1.000(Reference)	

Once	0.893 (0.527-1.515)	0.676
≥Twice	0.924 (0.482-1.771)	0.811
**Duration of inhaled medication use (years)**		
<1	1.000(Reference)	
1-5	1.411 (0.849-2.342)	0.184
>5	1.107 (0.584-2.098)	0.756
**Type of inhaled maintenance therapy**		
Triple therapy with LAMA-LABA-ICS	1.000(Reference)	
Dual therapy with LABA-ICS	2.110 (1.073-4.147)	0.030
Dual therapy with LAMA-LABA	1.342 (0.638-2.825)	0.439
Monotherapy with LAMA	1.719 (0.529-5.584)	0.368
Two separate inhalers	3.226 (0.966-10.769)	0.057
**Daily dosing frequency for inhaled medication**		
Once	1.000(Reference)	
Twice	0.753 (0.377-1.506)	0.423
**Medications prescribed for other chronic diseases**		
No	1.000(Reference)	
Yes	0.922 (0.593-1.434)	0.718
**Necessity beliefs**	0.723 (0.659-0.793)	<0.001
**Concerns beliefs**	1.057 (0.970-1.152)	0.209

Outcome: MARS-5 score <25. A total of 266 (59.4%) inpatients were non-adherent to inhaled medication. BMQ-Specific Necessity and Concerns variables were replaced by their continuous total scores. All other covariates were unchanged. Bold values indicated a p-value < 0.05. OR, odds ratio; CI, confidence interval.

Supplementary Table S3 Multivariable logistic regression analyses of factors associated with non-adherence to inhaled medication (Adjusted model after excluding 32 participants without pulmonary function results)

**Characteristics**	**Adjusted OR (95% CI)**	***P-value***
**Age (years)**		
<65	1.000(Reference)	
65-74	1.305 (0.768-2.217)	0.324
≥75	1.590 (0.850-2.975)	0.147
**Gender**		
Female	1.000(Reference)	
Male	1.017 (0.547-1.891)	0.958
**Smoking status**		
Never smoked before	1.000(Reference)	
Current smoker	1.364 (0.715-2.600)	0.346
Ex-smokers	0.736 (0.401-1.351)	0.322
**Education level**		
≤High school	1.000(Reference)	
>High school	0.883 (0.430-1.814)	0.734
**Occupation**		
Physical labor	1.000(Reference)	
Office work	0.879 (0.357-2.166)	0.779
Unemployed/Retired	0.608 (0.358-1.032)	0.065
**Income**		
Low	1.000(Reference)	
High	0.473 (0.242-0.926)	0.029
**Place of residence**		
Urban	1.000(Reference)	
Rural	0.931 (0.564-1.539)	0.782
**The number of hospital admissions due to exacerbations over the last year**		

0	1.000(Reference)	
Once	0.900 (0.523-1.551)	0.705
≥Twice	0.887 (0.451-1.743)	0.727
**Duration of inhaled medication use (years)**		
<1	1.000(Reference)	
1-5	1.367 (0.813-2.299)	0.239
>5	1.040 (0.547-1.977)	0.905
**Type of inhaled maintenance therapy**		
Triple therapy with LAMA-LABA-ICS	1.000(Reference)	
Dual therapy with LABA-ICS	1.981 (0.987-3.978)	0.055
Dual therapy with LAMA-LABA	1.519 (0.709-3.254)	0.282
Monotherapy with LAMA	1.764 (0.522-5.960)	0.361
Two separate inhalers	2.385 (0.692-8.212)	0.168
**Daily dosing frequency for inhaled medication**		
Once	1.000(Reference)	
Twice	0.838 (0.414-1.696)	0.622
**Medications prescribed for other chronic diseases**		
No	1.000(Reference)	
Yes	0.869 (0.554-1.365)	0.543
**Necessity beliefs**		
High	1.000(Reference)	
Low	4.916 (3.082-7.842)	<0.001
**Concerns beliefs**		
High	1.000(Reference)	
Low	0.729 (0.460-1.156)	0.180

Outcome: MARS-5 score <25. After excluding the 32 participants whose pulmonary function result was unavailable in the medical records, 416 participants remained. A total of 242 (58.2%) inpatients were non-adherent to inhaled medication. All other covariates were unchanged. Bold values indicated a p-value < 0.05. OR, odds ratio; CI, confidence interval.

Supplementary Table S4 Multivariable logistic regression analyses of factors associated with non-adherence to inhaled medication (Adjusted model using MARS-5 cut-off of 20 points)

**Characteristics**	**Adjusted OR (95% CI)**	***P-value***
**Age (years)**		
<65	1.000(Reference)	
65-74	0.531 (0.244-1.156)	0.111
≥75	1.043 (0.434-2.507)	0.925
**Gender**		
Female	1.000(Reference)	
Male	1.701 (0.696-4.157)	0.244
**Smoking status**		
Never smoked before	1.000(Reference)	
Current smoker	0.870 (0.337-2.246)	0.773
Ex-smokers	0.688 (0.277-1.710)	0.421
**Education level**		
≤High school	1.000(Reference)	
>High school	0.898 (0.255-3.169)	0.868
**Occupation**		
Physical labor	1.000(Reference)	
Office work	1.537 (0.314-7.528)	0.596
Unemployed/Retired	1.517 (0.719-3.202)	0.274
**Income**		
Low	1.000(Reference)	
High	0.416 (0.138-1.254)	0.119
**Place of residence**		
Urban	1.000(Reference)	
Rural	1.214 (0.581-2.537)	0.606
**The number of hospital admissions due to exacerbations over the last year**		
0	1.000(Reference)	

Once	1.254 (0.579-2.717)	0.566
≥Twice	0.714 (0.257-1.981)	0.518
**Duration of inhaled medication use (years)**		
<1	1.000(Reference)	
1-5	1.138 (0.549-2.360)	0.728
>5	1.109 (0.418-2.948)	0.835
**Type of inhaled maintenance therapy**		
Triple therapy with LAMA-LABA-ICS	1.000(Reference)	
Dual therapy with LABA-ICS	2.348 (0.706-7.806)	0.164
Dual therapy with LAMA-LABA	2.039 (0.480-8.665)	0.334
Monotherapy with LAMA	2.456 (0.403-14.963)	0.330
Two separate inhalers	5.360 (0.954-30.104)	0.057
**Daily dosing frequency for inhaled medication**		
Once	1.000(Reference)	
Twice	0.862 (0.275-2.702)	0.799
**Medications prescribed for other chronic diseases**		
No	1.000(Reference)	
Yes	1.040 (0.537-2.018)	0.906
**Necessity beliefs**		
High	1.000(Reference)	
Low	13.402 (5.373-33.430)	<0.001
**Concerns beliefs**		
High	1.000(Reference)	
Low	0.806 (0.417-1.558)	0.521

Outcome: MARS-5 score <20. When the alternative MARS-5 cut-off of 20 points was applied, 51 (11.4%) participants were classified as non-adherent. All other covariates were unchanged. Bold values indicated a p-value <0.05. OR, odds ratio; CI, confidence interval.
